# The Role of SGLT2 Inhibitors in Atherosclerosis: A Narrative Mini-Review

**DOI:** 10.3389/fphar.2021.751214

**Published:** 2021-11-05

**Authors:** Aurélie Pahud de Mortanges, Dante Salvador Jr., Markus Laimer, Taulant Muka, Matthias Wilhelm, Arjola Bano

**Affiliations:** ^1^ Faculty of Medicine, University of Bern, Bern, Switzerland; ^2^ Institute of Social and Preventive Medicine, University of Bern, Bern, Switzerland; ^3^ Department of Diabetes, Endocrinology, Nutritional Medicine, and Metabolism, Inselspital, Bern University Hospital, University of Bern, Bern, Switzerland; ^4^ Department of Cardiology, Inselspital, Bern University Hospital, University of Bern, Bern, Switzerland

**Keywords:** atherosclerotic cardiovascular disease, SGLT2-inhibitors, subclinical atherosclerosis, diabetes, review

## Abstract

**Objective:** Sodium glucose cotransporter 2 inhibitors (SGLT2-is) are antidiabetic drugs that improve glycemic control by limiting urinary glucose reuptake in the proximal tubule. SGLT2-is might suppress atherosclerotic processes and ameliorate the prognosis of patients with diabetes mellitus diagnosed with or at high risk of atherosclerotic cardiovascular disease (ASCVD). In this mini review, we examine the role of SGLT2-is in the development and progression of atherosclerosis throughout its spectrum, from subclinical atherosclerosis to ASCVD.

**Data Sources**—PubMed and Google Scholar were searched for publications related to SGLT2-is and atherosclerosis. All types of articles were considered, including clinical trials, animal studies, *in vitro* observations, and reviews and meta-analyses. Data were examined according to their impact and clinical relevance.

**Synopsis of Content**—We first review the underlying mechanisms of SGLT2-is on the development and progression of atherosclerosis, including favorable effects on lipid metabolism, reduction of systemic inflammation, and improvement of endothelial function. We then discuss the putative impact of SGLT2-is on the formation, composition, and stability of atherosclerotic plaque. Furthermore, we evaluate the effects of SGLT2-is in subclinical atherosclerosis assessed by carotid intima media thickness and pulse wave velocity. Subsequently, we summarize the effects of SGLT2-is in ASCVD events, including ischemic stroke, angina pectoris, myocardial infarction, revascularization, and peripheral artery disease, as well as major adverse cardiovascular events, cardiovascular mortality, heart failure, and chronic kidney disease. Moreover, we examine factors that could modify the role of SGLT2-is in atherosclerosis, including sex, age, diabetes, glycemic control, ASCVD, and SGLT2-i compounds. Additionally, we propose future directions that can improve our understanding of SGLT2-is and atherosclerosis.

## Introduction

Atherosclerosis is a progressive disease process, characterized by focal accumulations of lipids, complex carbohydrates, blood products, fibrous elements, and calcium deposits in the intima of arteries, which are also associated with medial changes (Lusis, 2000; Maurice et al., 2007). In its early stages, atherosclerosis remains clinically silent (i.e., subclinical atherosclerosis) and may further progress to atherosclerotic cardiovascular disease (ASCVD) and death (Ahmadi et al., 2019). In order to stop the progression of atherosclerosis and prevent cardiovascular (CV) events, it is essential to detect and manage it early on (Ahmadi et al., 2019). Besides lifestyle modifications, medications can help reduce progression of atherosclerosis. In addition to lipid- or blood pressure–lowering agents, the novel antidiabetic drugs sodium glucose cotransporter 2 inhibitors (SGLT2-is) might also suppress atherosclerotic processes and ameliorate the patients’ prognosis. SGLT2-is, including canagliflozin, dapagliflozin, and empagliflozin, improve glycemic control by inhibiting glucose reuptake in the proximal tubule and increasing renal glucose excretion (Clar et al., 2012). This narrative mini-review provides an overview on the role of SGLT2-is in the development and progression of atherosclerotic lesions and their possible effects on subclinical and clinical atherosclerosis.

## Methods

PubMed and Google Scholar were searched to identify relevant publications on SGLT2-is and atherosclerosis. Key words included the following: “SGLT2 inhibitor, canagliflozin, dapagliflozin, empagliflozin, ertugliflozin, ipragliflozin, luseogliflozin, remogliflozin, sotagliflozin, tofogliflozin, atherosclerosis, carotid intima media thickness, plaque, ankle-brachial index, coronary artery calcification score, pulse wave velocity, transient ischemic attack, ischemic stroke, coronary heart disease, angina pectoris, acute coronary syndrome, myocardial infarction, coronary revascularization, renal artery stenosis, and peripheral artery disease.” All types of articles were considered, including clinical trials, animal studies, *in vitro* observations, reviews, and meta-analyses. Since this is a narrative mini-review, we prioritized the most clinically relevant and up-to-date articles in the current literature.

## SGLT2 Inhibitors and the Pathogenesis of Atherosclerosis

**FIGURE 1:**
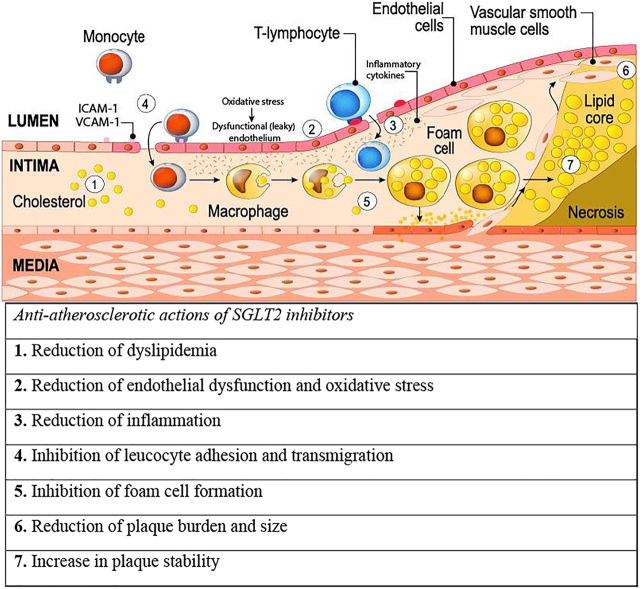
Putative mechanisms of action of SGLT2 inhibitors on pathways leading to atherosclerosis. 1–5 summarize effects of SGLT2 inhibitors against pathogenic processes that lead to development of atherosclerosis, while 6 and 7 summarize SGLT2 inhibitor effects against the progression and instability of atherosclerosis. Abbreviations: ICAM-1, intracellular adhesion molecule 1; SGLT2, sodium glucose cotransporter 2; VCAM-1, vascular cell adhesion molecule. Illustration adapted under standard license: designua/stock.adobe.com.

Several underlying mechanisms can explain the role of SLGT2-is in the pathogenesis of atherosclerosis (Figure 1; Table 1). SGLT2-is may prevent the development of atheroma by adjusting dyslipidemia, restoring normal endothelial function, reducing oxidative stress, decreasing inflammation, and inhibiting monocyte-macrophage-foam cell evolution (Terasaki et al., 2015; Leng et al., 2016; Han et al., 2017; Al-Sharea et al., 2018; Dimitriadis et al., 2019; Ganbaatar et al., 2020; Lee et al., 2020; Liu et al., 2021; Park et al., 2021). Furthermore, SGLT2-is may prevent the progression of atherosclerosis by reducing plaque size and burden, altering plaque composition, and improving plaque stability (Terasaki et al., 2015; Leng et al., 2016; Al-Sharea et al., 2018; Lee et al., 2020).

**TABLE 1:** SGLT2 inhibitors and pathogenesis of atherosclerosis.

Pathogenic process involved	Compound	Study (first author, year)	Type of study (e.g., human, animals, *in vitro*)	Results
Dyslipidemia	Dapagliflozin	Hayashi et al. (2017)	Patients with type 2 diabetes	↓Small dense LDL
	Canagliflozin	Kamijo et al. (2019)	Patients with type 2 diabetes	↑HDL
	Empagliflozin	Liu et al. (2021)	Normoglycemic mice	↓LDL, HDL unchanged
Endothelial dysfunction and oxidative stress	Dapagliflozin	Leng et al. (2016)	Diabetic mice	↓ROS production
	Dapagliflozin	Ganbaatar et al. (2020)	Diabetic mice	↓NADPH activity
	Empagliflozin	Cooper et al. (2019)	*In vitro* (human cells)	Restore and preserve glycocalyx, thus maintaining vascular health
	Empagliflozin	Liu et al. (2021)	Normoglycemic mice	↓Renin, aldosterone, norepinephrine, neuropeptide Y
	Empagliflozin	Park et al. (2021)	*In vitro* (porcine cells)	Reversed the upregulation of endothelial cell senescence genes, reversed the downregulation of eNOS and NO
Inflammation	Dapagliflozin	Leng et al. (2016)	Diabetic mice	↓IL-1B, IL-18, and NLRP3 inflammasome activation
	Canagliflozin	Lee et al. (2020)	Normoglycemic rabbits	↓IL-1B, IL-6, TNF-α expression
	Canagliflozin	Heerspink et al. (2019)	*In vitro* (human cells)	↓TNFR1, IL-6
	Empagliflozin	Han et al. (2017)	Hypercholesterolemic mice	↓Serum MCP-1, TNF- α, IL-6
	Empagliflozin	Dimitriadis et al. (2019)	Hypercholesterolemic mice	↓MCP-1 mRNA expression, but not MCP-1 proteins in lesions
	Empagliflozin	Ganbaatar et al. (2020)	Diabetic mice	↓MCP-1
	Empagliflozin	Liu et al. (2021)	Normoglycemic mice	↓IL-10, but not IL-1β and IL-6
Leucocyte adhesion and transmigration	Canagliflozin	Nasiri-Ansari et al. (2018)	Atherosclerotic mice	↓VCAM-1
	Empagliflozin	Ganbaatar et al. (2020)	Diabetic mice	↓VCAM-1
	Empagliflozin	Pennig et al. (2019)	Diabetic mice	↓Monocyte adhesion on the endothelial wall
	Luseogliflozin	Nakatsu et al. (2017)	Diabetic mice	Reversed upregulation of ICAM-1
Foam cell formation	Dapagliflozin	Terasaki et al. (2015)	Diabetic mice	↓Cholesterol ester accumulation in macrophages
	Dapagliflozin	Lee et al. (2020)	Normoglycemic rabbit	↓Macrophage infiltration and polarization
	Empagliflozin	Ganbaatar et al. (2020)	Diabetic mice	↓Macrophage accumulation
	Ipragliflozin	Terasaki et al. (2015)	Diabetic mice	↓Cholesterol ester accumulation in macrophages
Plaque burden and size	Dapagliflozin	Terasaki et al. (2015)	Diabetic mice	↓Atheroma size
	Dapagliflozin	Lee et al. (2020)	Normoglycemic rabbit	↓Atheroma burden
	Dapagliflozin	Leng et al. (2016)	Diabetic mice	↓Atherosclerotic lesion size
	Empagliflozin	Han et al. (2017)	Hypercholesterolemic mice	↓Atheroma size
	Empagliflozin	Dimitriadis et al. (2019)	Hypercholesterolemic mice	Attenuated progression of atherosclerosis
	Empagliflozin	Pennig et al. (2019)	Diabetic mice	↓Atherosclerotic lesion size
	Empagliflozin	Ganbaatar et al. (2020)	Diabetic mice	↓Atherosclerotic lesion size
	Empagliflozin	Liu et al. (2021)	Normoglycemic mice	↓Atherosclerotic lesion size
	Luseogliflozin	Nakatsu et al. (2017)	Diabetic mice	Attenuated progression of atherosclerosis
Plaque composition and stability	Dapagliflozin	Terasaki et al. (2015)	Diabetic mice	↓Macrophage infiltration of plaques
	Dapagliflozin	Leng et al. (2016)	Diabetic mice	↓Cholesterol crystals in lesions
	Dapagliflozin	Lee et al. (2020)	Normoglycemic rabbit	↓Lipid accumulation in lesions
	Dapagliflozin	Spigoni et al. (2020)	*In vitro* (human cells)	Plaque stabilization and thrombosis inhibition through reduction of lipotoxic damage and inhibition of platelet activation
	Empagliflozin	Spigoni et al. (2020)	*In vitro* (human cells)	Plaque stabilization and thrombosis inhibition through reduction of lipotoxic damage and inhibition of platelet activation
	Luseogliflozin	Nakatsu et al. (2017)	Diabetic mice	↓Lipid accumulation in lesions

For each pathogenic process, evidence from the most relevant studies are reported, providing details on study population and relevant findings on specific pathophysiological mechanisms.

Abbreviations: eNOS, endothelial nitric oxide synthase; HDL, high density lipoprotein; ICAM-1, intracellular adhesion molecule 1; IL, interleukin; LDL, low density lipoprotein; MCP-1, monocyte chemoattractant protein; mRNA, messenger ribonucleic acid; NADPH, nicotinamide adenine dinucleotide phosphate; NLRP3, nucleotide-binding oligomerization domain-like receptor, leucine-rich repeat, pyrin domain-containing 3; NO, nitric oxide; ROS, reactive oxygen species; SGLT, sodium glucose cotransporter; TNF, tumor necrosis factor; TNFR1, tumor necrosis factor receptor 1; VCAM-1, vascular cell adhesion molecule.

### Dyslipidemia

Excess cholesterol substrates can increase the susceptibility of arterial walls to atherosclerosis. Several studies in animals and humans have shown that SGLT2-is reduce serum total cholesterol and triglyceride levels (Calapkulu et al., 2019; Ganbaatar et al., 2020; Liu et al., 2021). In patients with diabetes, dapagliflozin reduced the potent atherogenic particles of low-density lipoprotein (LDL) (Hayashi et al., 2017), while canagliflozin increased high-density lipoprotein (HDL) levels (Kamijo et al., 2019). In normoglycemic mice models, empagliflozin reduced LDL, but no changes in HDL levels were observed (Liu et al., 2021).

### Endothelial Dysfunction and Oxidative Stress

SLGT2-is can reduce endothelial dysfunction directly by affecting endothelial cells or indirectly by reducing oxidative stress and sympathetic activation. Hence, SGLT2-is reverse the upregulation of endothelial cell senescence genes, further reducing the predisposition to endothelial dysfunction (Park et al., 2021). In diabetic mice, SGLT2-is manifested antioxidant effects by reducing reactive oxygen species (ROS) production and by reversing the increased NADPH activity (Leng et al., 2016; Ganbaatar et al., 2020). Empagliflozin also restored and preserved the glycocalyx of human abdominal aortic endothelial cells, resulting in maintained vascular health (Cooper et al., 2019). Oxidative stress was also reversed by empagliflozin in porcine endothelial cells, through inhibition of nitric oxide formation (Park et al., 2021). Additionally, the administration of empagliflozin in normoglycemic mice reduced renin, aldosterone, norepinephrine, and neuropeptide Y (Liu et al., 2021).

### Inflammation

SGLT2-is can affect inflammatory cytokines that promote activation and migration of monocytes into the tunica intima in both diabetic and normoglycemic models (Leng et al., 2016; Han et al., 2017; Dimitriadis et al., 2019; Ganbaatar et al., 2020; Lee et al., 2020; Liu et al., 2021). Empagliflozin reduced monocyte chemoattractant protein (MCP)-1, interleukin (IL)-6, IL-10, and tumor necrosis factor (TNF)-α in both normoglycemic and diabetic mice (Han et al., 2017; Dimitriadis et al., 2019; Ganbaatar et al., 2020; Liu et al., 2021). Dapagliflozin reduced IL-1β, IL-18, and NLRP3 inflammasome activation in diabetic mice (Leng et al., 2016). Canagliflozin reduced IL-1β, IL-6, and TNF-α expression in normoglycemic rabbits (Lee et al., 2020). An *in vitro* study on human plasma samples also suggested that canagliflozin contributes to the reduction of several inflammatory biomarkers (Heerspink et al., 2019).

### Leukocyte Adhesion and Transmigration

SGLT2-is can reduce leukocyte adhesion to endothelial cells and transmigration into the intra-intimal space. The adhesion of leukocytes on endothelial surfaces is facilitated by the endothelial adhesion molecules such as vascular cell adhesion molecule (VCAM)-1 and intracellular adhesion molecule (ICAM)-1, with ICAM-1 also facilitating monocyte transmigration (Oppenheimer-Marks et al., 1991; Ley and Huo, 2001; Muller, 2002). In diabetic mice, the upregulation of ICAM-1 was reversed by luseogliflozin (Nakatsu et al., 2017), whereas empagliflozin reduced VCAM-1 (Ganbaatar et al., 2020) and additionally decreased adhesion of proinflammatory monocytes on the endothelial wall (Pennig et al., 2019). These results are also in line with those of a study in an atherosclerotic mouse model, showing a reduction in VCAM-1 by canagliflozin (Nasiri-Ansari et al., 2018).

### Foam Cell Formation

The excessive lipid accumulation triggers the transition of macrophages into foam cells, which are key cellular precursors of atheromas (Kang et al., 2020). In diabetic mice models, dapagliflozin, empagliflozin, and ipragliflozin reduced macrophage proliferation, infiltration, and formation of cholesterol esters that are associated with the degree of foam cell formation (Terasaki et al., 2015; Ganbaatar et al., 2020). Additionally, dapagliflozin reduced macrophage infiltration and polarization in a normoglycemic rabbit model (Lee et al., 2020).

### Plaque Burden and Size

SGLT2-is can reduce atheroma burden and plaque size (Terasaki et al., 2015; Leng et al., 2016; Al-Sharea et al., 2018). In normoglycemic and diabetic animal models of atherosclerosis, dapagliflozin, empagliflozin, and luseogliflozin reduced the number of atheroma plaques, atherosclerotic lesion size and surface area (Terasaki et al., 2015; Leng et al., 2016; Han et al., 2017; Nakatsu et al., 2017; Dimitriadis et al., 2019; Pennig et al., 2019; Ganbaatar et al., 2020; Lee et al., 2020; Liu et al., 2021). On the other hand, canagliflozin did not reduce plaque size in normoglycemic mice, suggesting that it may abate atherosclerosis progression only in the presence of pronounced hyperglycaemia (Day et al., 2020).

### Plaque Composition and Stability

Atherosclerotic plaques cause arterial stenosis and may eventually rupture. The stability of atherosclerotic plaques decreases when plaques have an increased lipid content, increased quantity of foam cells, and increased amount of matrix metalloproteinases (Johnson et al., 2006; Bentzon et al., 2014). Dapagliflozin reduced macrophages and cholesterol crystal content in the atherosclerotic plaques of diabetic mice (Terasaki et al., 2015; Leng et al., 2016). In human myeloid angiogenic cells, dapagliflozin and empagliflozin reduced lipotoxic damage and platelet activation, which may contribute to plaque stabilization and thrombosis inhibition (Spigoni et al., 2020). Moreover, luseogliflozin decreased the amount of matrix metalloproteinases in diabetic mice (Nakatsu et al., 2017). In normoglycemic rabbits, dapagliflozin reduced lipid accumulation within atherosclerotic plaques (Lee et al., 2020).

### Indirect Mechanisms

SGLT2-is may indirectly reduce the risk of atherosclerotic processes *via* several beneficial CV effects, including reduction in blood pressure, body weight, and epicardial fat volume (Storgaard et al., 2016; Verma and McMurray, 2018). *Via* natriuretic and osmotic diuretic properties, SGLT2-is reduce the preload and afterload, which further results in decreased blood pressure and reduction of atherosclerosis (Verma and McMurray, 2018). SGLT2-is also reduce body weight through renal excretion of glucose and corresponding calories (Storgaard et al., 2016; Pereira and Eriksson, 2019). Moreover, the administration of SGLT2-is in patients with diabetes and coronary artery disease can decrease epicardial fat volume, most likely by reductions in body weight and inflammation markers (Sato et al., 2018; Verma and McMurray, 2018).

## SGLT2 Inhibitors and Subclinical Atherosclerosis

Several noninvasive measures of subclinical atherosclerosis, including carotid intima media thickness (cIMT) and pulse wave velocity (PWV), are used to quantify the atherosclerotic burden in asymptomatic individuals and are predictive for future CV events (Folsom et al., 2008; Van Bortel et al., 2012; Zhong et al., 2018; Ahmadi et al., 2019). The UTOPIA study, a prospective, randomized, open-label, parallel-design trial performed in 340 subjects with type 2 diabetes mellitus (T2DM) and no history of CV disease, investigated whether tofogliflozin has favorable effects on cIMT compared to conventional treatment using drugs other than SGLT2-is (Katakami et al., 2020). After 104 weeks, tofogliflozin reduced the common mean cIMT compared to the baseline (mean change ±standard error, −0.132 ± 0.007, *p* < 0.001), but there was no significant difference between the tofogliflozin and the conventional treatment group. In a prespecified subanalysis of the UTOPIA trial, a significant attenuation of PWV was found in the tofogliflozin group compared to the conventional treatment group (mean change,-104.7 cm/s; 95% confidence interval [95%CI],−177 to −32.4) (Katakami et al., 2021). Another prospective, randomized study in 160 patients with T2DM also found significantly improved PWV in patients treated with SGLT2-is compared to those treated with basal insulin after a 12-month treatment period (Ikonomidis et al., 2020). Overall, the current evidence regarding the potential influence of SGLT2-is on surrogate measures of subclinical atherosclerosis is limited, due to the restricted number of existing trials, relatively small sample sizes of previous studies, the relatively short follow-up time, potential differences in baseline characteristics between the treatment and control groups, and inter-sonographer variability of measurements. Further studies may also consider assessing the coronary artery calcification score (CAC), which is superior to other measures of subclinical atherosclerosis in the prediction of future CV events (Folsom et al., 2008).

## SGLT2 Inhibitors and Clinical Atherosclerosis

ASCVD comprises acute coronary syndromes (myocardial infarction [MI] or unstable angina), stable angina, stroke, transient ischemic attack (TIA), arterial revascularization, and peripheral artery disease (PAD) (Grundy et al.; ADA, 2016; Mach et al., 2020). Major adverse cardiovascular events (MACEs), CV mortality, heart failure (HF), and chronic kidney disease (CKD) are greatly driven by atherosclerosis and therefore will also be discussed below. Given the large body of evidence on SGLT2-is and clinical atherosclerosis, this section mainly reports the results of previous systematic reviews and meta-analyses.

### Major Adverse Cardiovascular Events, Cardiovascular Mortality, Heart Failure, and Chronic Kidney Disease

Previous studies have consistently shown beneficial effects of SGLT2-is in reducing the risk of MACEs, CV mortality, HF, and CKD (Table 2). Although these diseases are commonly of atherosclerotic origin, we acknowledge that they can also be influenced by mechanisms other than atherosclerosis (Hupfeld and Mudaliar, 2019). In particular, HF may occur in the form of “diabetic cardiomyopathy” that is characterized by ventricular dysfunction in a patient with diabetes in the absence of coronary artery disease or hypertension (Hupfeld and Mudaliar, 2019).

**TABLE 2:** Summary of selected studies investigating the effects of SGLT2 inhibitors on ASCVD events.

	Outcome		Study	Study type^a^	Population size relevant to outcome (patients in the treatment group plus patients in the control group)	Control group^b^	Glycemic status^c^	HR/RR/OR (95% CI), compared to controls^d^
SGLT2-is								
Any SGLT2-is	MACE		Zhu et al. (2020)	Umbrella review of MAs of RCTs	55,283	Mixed	Mixed	RR: 0.87 (0.82–0.93)
			Ghosh-Swaby et al. (2020)	MA of CVOTs	38,723	Standard care or placebo	With or at risk of T2DM	RR: 0.88 (0.82–0.94)
			McGuire et al. (2021)	MA of CVOTs	46,969	Placebo	T2DM	HR: 0.90 (0.85–0.95)
	Cardiovascular mortality		Zhu et al. (2020)	Umbrella review of MAs of RCTs	61,266	Mixed	Mixed	RR: 0.82 (0.75–0.90)
			Zheng et al. (2018)	Network MA of RCTs	69,276	Placebo or no treatment	T2DM	HR: 0.79 (0.69–0.91)
			McGuire et al. (2021)	MA of CVOTs	46,969	Placebo	T2DM	HR: 0.85 (0.78–0.93)
	HF	HF	Zhu et al. (2020)	Umbrella review of MAs of RCTs	51,348	Mixed	Mixed	RR: 0.68 (0.63–0.73)
		HF	Zheng et al. (2018)	Network MA of RCTs	64,351	Placebo or no treatment	T2DM	HR: 0.62 (0.54–0.72)
		HHF	McGuire et al. (2021)	MA of CVOTs	46,969	Placebo	T2DM	HR: 0.68 (0.61–0.76)
	ESKD		Neuen et al. (2019)	MA of CVOT	38,723	Placebo	T2DM	RR 0.65 (0.53–0.81)
	MI		Zhu et al. (2020)	Umbrella review of MAs of RCTs	59,640	Mixed	Mixed	RR: 0.86 (0.78–0.94)
			Zheng et al. (2018)	Network MA of RCTs	73,057	Placebo or no treatment	T2DM	HR: 0.86 (0.77–0.97)
	Unstable AP		Zhu et al. (2020)	Umbrella review of MAs of RCTs	18,389	Mixed	Mixed	RR: 0.95 (0.72–1.25)
			Zheng et al. (2018)	Network MA of RCTs	46,237	Placebo or no treatment	T2DM	HR: 0.97 (0.74–1.27)
	Stroke		Zhu et al. (2020)	Umbrella review of MAs of RCTs	68,046	Mixed	Mixed	RR: 0.96 (0.85–1.08)
			Zheng et al. (2018)	Network MA of RCTs	61,345	Placebo or no treatment	T2DM	HR: 0.92 (0.79–1.08)
	PAD		Dicembrini et al. (2019)	MA of RCTs	50,963	Placebo or other active comparators different from SGLT2-is	T2DM	OR: 1.20 (0.99–1.44)
Canagliflozin	MACE		Zhu et al. (2020)	Umbrella review of MAs of RCTs	NA	Mixed	Mixed	RR: 0.84 (0.75–0.93)
	Cardiovascular mortality		Zhu et al. (2020)	Umbrella review of MAs of RCTs	22,778	Mixed	Mixed	RR: 0.82 (0.71–0.96)
	HF		Zhu et al. (2020)	Umbrella review of MAs of RCTs	NA	Mixed	Mixed	RR: 0.65 (0.54–0.78)
	ESKD		Neuen et al. (2019)	MA referring to the CANVAS Program	10,142	Placebo	T2DM	HR: 0.77 (0.30–1.97)
			Neuen et al. (2019)	MA referring to CREDENCE	4,401	Placebo	T2DM	HR: 0.68 (0.54–0.86)
	MI		Zhu et al. (2020)	Umbrella review of MAs of RCTs	19,459	Mixed	Mixed	RR: 0.86 (0.73–1.02)
	Unstable AP		Zhu et al. (2020)	Umbrella review of MAs of RCTs	NA	Mixed	Mixed	RR: 0.66 (0.17–2.50)
	Stroke		Zhu et al. (2020)	Umbrella review of MAs of RCTs	20,712	Mixed	Mixed	RR: 0.86 (0.71–1.03)
	PAD		Dicembrini et al. (2019)	MA of RCTs	14,594	Placebo or other active comparators different from SGLT2-is	T2DM	OR: 1.80 (1.28–2.54)
Dapagliflozin	MACE		Zhu et al. (2020)	Umbrella review of MAs of RCTs	25,679	Mixed	Mixed	RR: 0.92 (0.83–1.01)
	Cardiovascular mortality		Zhu et al. (2020)	Umbrella review of MAs of RCTs	27,929	Mixed	Mixed	RR: 0.89 (0.77–1.02)
	HF		Zhu et al. (2020)	Umbrella review of MAs of RCTs	26,260	Mixed	Mixed	RR: 0.70 (0.60–0.82)
	ESKD		Neuen et al. (2019)	MA referring to DECLARE-TIMI 58	17,160	Placebo	T2DM	HR: 0.31 (0.13–0.79)
			Heerspink et al. (2020)	Kidney-specific outcome trial (DAPA-CKD)	2,152	Placebo	Mixed	HR: 0.64 (0.50–0.82)
	MI		Zhu et al. (2020)	Umbrella review of MAs of RCTs	25,418	Mixed	Mixed	RR: 0.77 (0.51–1.16)
	Unstable AP		Zhu et al. (2020)	Umbrella review of MAs of RCTs	7,289	Mixed	Mixed	RR: 0.87 (0.47–1.59)
	Stroke		Zhu et al. (2020)	Umbrella review of MAs of RCTs	23,799	Mixed	Mixed	RR: 1.01 (0.85–1.20)
	PAD	PAD	Dicembrini et al. (2019)	MA of RCTs	21,586	Placebo or other active comparators different from SGLT2-is	T2DM	OR: 0.97 (0.74–1.29)
		Ischemic limb event	Bonaca et al. (2020)	CVOT (DECLARE-TIMI 58)	17,160	Placebo	T2DM	HR: 1.07 (0.90–1.26)
	Lower-extremity revascularization		Bonaca et al. (2020)	CVOT (DECLARE-TIMI 58)	17,160	Placebo	T2DM	HR: 1.00 (0.81–1.24)
Empagliflozin	MACE		Zhu et al. (2020)	Umbrella review of MAs of RCTs	18,312	Mixed	Mixed	RR: 0.85 (0.77–0.94)
	Cardiovascular mortality		Zhu et al. (2020)	Umbrella review of MAs of RCTs	12,309	Mixed	Mixed	RR: 0.62 (0.50–0.78)
	HF		Zhu et al. (2020)	Umbrella review of MAs of RCTs	18,312	Mixed	Mixed	RR: 0.64 (0.53–0.77)
	ESKD		Neuen et al. (2019)	MA referring to EMPA-REG OUTCOME	7,020	Placebo	T2DM	RR: 0.60 (0.18–1.98)
	MI		Zhu et al. (2020)	Umbrella review of MAs of RCTs	15,750	Mixed	Mixed	RR: 0.84 (0.68–1.04)
	Unstable AP		Zhu et al. (2020)	Umbrella review of MAs of RCTs	18,312	Mixed	Mixed	RR: 0.96 (0.78–1.18)
	Stroke		Zhu et al. (2020)	Umbrella review of MAs of RCTs	16,744	Mixed	Mixed	RR: 1.11 (0.86–1.43)
	PAD		Dicembrini et al. (2019)	MA of RCTs	14,319	Placebo or other active comparators different from SGLT2-is	T2DM	OR: 1.09 (0.76–1.56)
	Coronary revascularization		Zinman et al. (2015)	CVOT (EMPA-REG OUTCOME)	7,020	Placebo	T2DM	HR: 0.86 (0.72–1.04)

a For each outcome, recent meta-analyses of RCTs are preferentially reported. In case no meta-analyses were available, individual RCTs or other study types were reported.

b In the column “Control group”, the term “mixed” indicates that the comparator includes a lifestyle intervention, no treatment, placebo or other glucose-lowering medications.

c In the column “Glycemic status”, the term “mixed” indicates a mix of patients with diabetes, pre-diabetes, or at high risk of diabetes. The definition of pre-diabetes or high risk for diabetes was blood glucose concentration below the cut-off for diabetes, but higher than is considered normal, such as isolated impairment of fasting glucose, glucose tolerance, HbA₁c, or combinations thereof.

d In the study by Zhu et al., 2020, the quality of evidence was graded as high for all outcomes, except for:—moderate quality of evidence for, MI and stroke in canagliflozin and empagliflozin, and MI in dapagliflozin —very low quality of evidence for unstable AP in any SGLT2-is, canagliflozin, and dapagliflozin.

Abbreviations: AP, angina pectoris; ASCVD, atherosclerotic cardiovascular disease; CI, confidence interval; CVOT, cardiovascular outcome trial; ESKD, end stage kidney disease; HF, heart failure; HHF, hospitalization for heart failure; HR, hazard ratio; MA, meta-analysis; MAs, meta-analyses; MI, myocardial infarction; OR, odds ratio; PAD, peripheral artery disease; RCT, randomized controlled trial; RR, relative risk; SGLT2-i, sodium glucose cotransporter 2 inhibitor; T2DM, type 2 diabetes mellitus; NA, not available.

MACE. Although its definitions vary across studies, MACE is commonly defined as a three-point composite of CV mortality, nonfatal MI, and nonfatal stroke. An umbrella review of meta-analyses of 32 trials including a total of 55,283 participants with T2DM, pre-diabetes, or high risk of diabetes indicated that SGLT2-is are associated with a 13% risk reduction in MACE (relative risk [RR], 0.87; 95%CI, 0.82–0.93) (Zhu et al., 2020). In line, another meta-analysis with a total of 38,723 participants, including four cardiovascular outcome trials (CVOTs), namely, EMPA-REG OUTCOME, CANVAS, DECLARE-TIMI 58, and CREDENCE, showed a 12% lower risk of atherosclerotic MACE in the SGLT2-i group compared with the standard of care or placebo [hazard ratio (HR), 0.88; 95%CI, 0.82–0.94] (Ghosh-Swaby et al., 2020).

CV mortality. Two meta-analyses revealed an 18% (RR, 0.82; 95%CI, 0.75–0.90) and 21% risk reduction (HR, 0.79; 95%CI, 0.69–0.91) of CV mortality in SGLT2-i users compared to controls, respectively (Zheng et al., 2018; Zhu et al., 2020).

HF. A meta-analysis including 17 studies with a total of 51,348 participants with T2DM, pre-diabetes, or high risk of diabetes reported a 32% reduction of HF risk (RR, 0.68; 95%CI, 0.63–0.73) in SGLT2-i users (Zhu et al., 2020). Another meta-analysis of randomized controlled trials (RCTs) including 64,351 patients with diabetes found a 38% risk reduction for HF (HR, 0.62; 95%CI, 0.54–0.72) (Zheng et al., 2018).

CKD. A meta-analysis of four CVOTs (EMPA-REG OUTCOME, CANVAS, CREDENCE, and DECLARE-TIMI 58) including a total of 38,723 participants with diabetes found a 35% risk reduction of end-stage kidney disease (RR, 0.65; 95%CI, 0.53–0.81) in SGLT2-i users (Neuen et al., 2019).

### Distinct Atherosclerotic Cardiovascular Disease Events

Current evidence suggests that SGLT2-is reduce the risk of MI but do not reduce the risk of unstable angina, stroke, TIA, arterial revascularization, and PAD (Table 2).

MI and angina pectoris. Analyzing 40 trials with approximately 60,000 participants, an umbrella review showed that SGLT2-i users have a 14% lower risk of incident MI compared to controls (HR, 0.86; 95%CI, 0.78–0.94) (Zhu et al., 2020). These results were consistent with another meta-analysis of RCTs in patients with diabetes (Zheng et al., 2018). However, the aforementioned meta-analyses did not show differences in the risk of unstable angina between SGLT2-i users and controls (Zheng et al., 2018; Zhu et al., 2020).

Stroke and TIA. Several meta-analyses and reviews reported that SGLT2-is, including canagliflozin, dapagliflozin, and empagliflozin, do not affect the risk of stroke (Milonas and Tziomalos, 2018; Zheng et al., 2018; Sinha and Ghosal, 2019b; Sinha and Ghosal, 2019a; Zhu et al., 2020). In line, the empagliflozin and placebo arms in the EMPA-REG OUTCOME trial did not differ in the risk of TIA (Zinman et al., 2015). Further investigations evaluating the role of SGLT2-is in ischemic stroke are needed.

Arterial revascularization. A limited number of studies have investigated the likelihood of arterial revascularization among SGLT2-i users. In the EMPA-REG OUTCOME trial on empagliflozin and in the DECLARE-TIMI 58 trial on dapagliflozin, the administration of SGLT2-is did not affect the risks of coronary revascularization or lower extremity revascularization, respectively (Zinman et al., 2015; Bonaca et al., 2020).

PAD. There is inconsistent evidence regarding the role of SGLT2-is on PAD risk and subsequent lower limb amputations. Patients with diabetes are already at high risk of PAD, and SGLT2-is can presumably increase the risk of PAD even further *via* promoting glucosuria, volume depletion, and haemoconcentration (Shah et al., 2012). The CANVAS Program found increased risk of lower extremity amputations in the canagliflozin group compared to the placebo (HR, 1.97; 95%CI, 1.41–2.75) (Neal et al., 2017). However, the effects of canagliflozin on PAD may not be generalizable to other SGLT2-i compounds. In meta-analyses, dapagliflozin and empagliflozin were not associated with increased risk of amputations (Dicembrini et al., 2019; Heyward et al., 2020; Huang and Lee, 2020). Further studies are warranted to compare the risk of PAD between users of canagliflozin and users of other SGLT2-i compounds.

## SGLT2 Inhibitors and Atherosclerosis: Potential Effect Modifiers

Various factors including sex, age, diabetes, glycemic control, ASCVD, and SGLT2-i compounds can modify the association of SGLT2-is with atherosclerosis. The identification of effect modifiers is important, as it can help explain heterogeneity, improve current recommendations, and foster personalized treatment.

Sex. Men and women might have different responses to SGLT2-is, possibly due to differences in drug pharmacokinetics, pharmacodynamics, and adherence. However, a meta-analysis of four RCTs in patients with T2DM (EMPA-REG OUTCOME, CANVAS, DECLARE-TIMI 58, and CREDENCE) suggested that SGLT2-is may have comparable effects in men and women (Rådholm et al., 2020). Overall, evidence on sex differences remains inconclusive, given that women are often underrepresented in RCTs of SGLT2-is (O’Donoghue et al., 2021). In the CANVAS study on canagliflozin and the DECLARE-TIMI 58 study on dapagliflozin, less than 38% of participants were women (Neal et al., 2017; Wiviott et al., 2019). Future studies should include comparable proportions of men and women, which can allow an adequate assessment of possible sex differences in the safety and efficacy of SGLT2-is.

Age. Aging can alter the effects of SGLT2-is *via* affecting drug metabolism. Increasing age is associated with a gradual reduction in the glomerular filtration rate, which may downregulate the tubular expression of SGLT2 and further decline the glucose lowering effect of SGLT2-is (Cintra et al., 2019). Aging is also associated with decreased insulin sensitivity, sarcopenia, weight gain, and elevated adiposity, which influence the risk of hypoglycemia and could further affect the effectiveness of SGLT2-is (Cintra et al., 2019). Therefore, it would be of major importance to assess whether the potential anti-atherosclerotic properties of SGLT2-is differ by age. However, the recruitment of older participants is usually challenging. Further studies need to perform predefined analyses accounting for age.

Diabetes and glycemic control. Given that the main indication for SGLT2-is is the reduction of blood glucose, most clinical studies evaluating the role of SGLT2-is in ASCVD are conducted in patients with T2DM. However, SGLT2-is can also have beneficial effects in normoglycemic individuals. This assumption is supported by a meta-analysis of two RCTs (DAPA-HF for dapagliflozin and EMPEROR-Reduced for empagliflozin) in patients with HF, which showed that SGLT2-i users with and without diabetes have a similar reduction in the risk of a composite endpoint consisting of hospitalization for HF (HHF) and CV death (Zannad et al., 2020). Furthermore, the EMPA-REG OUTCOME trial including patients with T2DM and cardiovascular disease suggested that the benefits of empagliflozin in reducing the risk of HHF and CV death are independent of glycemic control (Inzucchi et al., 2018). Similarly, the DAPA-CKD study performed in patients with CKD suggested protective effects of SGLT2-is on the kidney, regardless of the presence or absence of diabetes (Heerspink et al., 2020). Future studies are needed to evaluate whether diabetes status, duration, and glycemic control modify the effects of SGLT2-is on distinct ASCVD events.

ASCVD. In RCTs of SGLT2-is, the cardiovascular risk profile of eligible participants varies according to the study inclusion criteria. Some RCTs (EMPA-REG-OUTCOME and VERTIS) included only patients with pre-existent ASCVD, whereas other RCTs (CANVAS, DECLARE-TIMI 58, and CREDENCE) included participants with and without ASCVD (Zinman et al., 2015; Neal et al., 2017; Perkovic et al., 2019; Wiviott et al., 2019; Cannon et al., 2020). A recent meta-analysis of RCTs in patients with diabetes concluded that the presence or absence of ASCVD does not modify the association of SGLT2-is with MACEs, CV deaths, and HHF, respectively (McGuire et al., 2021). Further stratified analyses by CV risk factors (e.g., hypertension and obesity) are warranted.

SGLT2-i compounds. Various SGLT2-i compounds have different selectivities for the SGLT2 receptor; thereby, effect differences may exist across compounds even within the SGLT2-i class. A meta-analysis of RCTs in patients with diabetes suggested that empagliflozin is associated with reduced risks of CV death and MACE and canagliflozin is associated with reduced risk of MACEs, while all analyzed SGLT2-is, including empagliflozin, canagliflozin, dapagliflozin, and ertugliflozin, are associated with reduced risk of HHF (McGuire et al., 2021).

## Conclusion and Future Directions

SGLT2-is can exert anti-atherosclerotic properties *via* affecting various pathways of atherogenesis, including dyslipidemia, endothelial dysfunction, oxidative stress, inflammation, leucocyte adhesion and transmigration, plaque composition and instability. Still, the exact underlying mechanisms linking SGLT2-is to atherosclerotic processes are yet to be fully elucidated. Furthermore, SGLT2-is have manifested beneficial effects in reducing the risk of MACEs, CV mortality, HF, and CKD, which are largely of atherosclerotic origin. However, the role of SGLT2-is in distinct ASCVD events remains to be explored more extensively. Current evidence supports that SGLT2-is can reduce the risk of MI, while the risks of unstable angina, stroke, TIA, arterial revascularization, and PAD seem to be unaffected by SGLT2-is. Further experimental and observational studies of high quality, with adequate number of events and follow-up time, need to examine the potential role of SGLT2-is in subclinical atherosclerosis and ASCVD events, not only in those with diabetes and pre-diabetes but also in normoglycemic individuals. The effects of SGLT2-i compounds need to be compared with one another and with those of other antidiabetic medications. Potential effect modification by age, sex, and comorbidities needs to be extensively explored.
